# Effects of Artesunate on Parasite Recrudescence and Dormancy in the Rodent Malaria Model *Plasmodium vinckei*

**DOI:** 10.1371/journal.pone.0026689

**Published:** 2011-10-24

**Authors:** Alexis N. LaCrue, Misty Scheel, Katherine Kennedy, Nikesh Kumar, Dennis E. Kyle

**Affiliations:** Department of Global Health, University of South Florida, Tampa, Florida, United States of America; Kenya Medical Research Institute - Wellcome Trust Research Programme, Kenya

## Abstract

Artemisinin (ART) is the recommended first line therapy for treating uncomplicated and drug-resistant *Plasmodium falciparum*, the most pathogenic form of malaria. However, treatment failure following ART monotherapy is not uncommon and resistance to this rapidly acting drug has been reported in the Thai-Cambodian border. Recent in vitro studies have shown that following treatment with dihydroartemisinin (DHA), the development of ring-stage parasites is arrested for up to 20 days. These arrested (*i.e.* dormant) rings could be responsible for the recrudescence of infection that is observed following ART monotherapy. To develop a better understanding of the stage-specific effects of ART and determine if dormancy occurs in vivo, the ART derivative artesunate (AS) was used to treat mice infected with the synchronous rodent malaria parasites *P. vinckei petteri* (non-lethal) and *P. v. vinckei* (lethal). Results show that in both the non-lethal and lethal strains, ring-stage parasites are the least susceptible to treatment with AS and that the day of treatment has more of an impact on recrudescence than the total dose administered. Additionally, 24 hrs post-treatment with AS, dormant forms similar in morphology to those seen in vitro were observed. Finally, rate of recrudescence studies suggest that there is a positive correlation between the number of dormant parasites present and when recrudescence occurs in the vertebrate host. Collectively, these data suggest that dormancy occurs in vivo and contributes to recrudescence that is observed following AS treatment. It is possible that this may represent a novel mechanism of parasite survival following treatment with AS.

## Introduction

Malaria is a mosquito-borne disease caused by a protozoan parasite from the genus *Plasmodium*. There are more than 100 species of *Plasmodium* that are transmitted by the bite of an infected female *Anopheles* mosquito, but only five are known to infect humans: *P. falciparium, P. vivax, P. ovale, P. malariae, and P. knowlesi*. Malaria is especially dangerous to pregnant women and small children, and in endemic countries, it is an important determinant of perinatal mortality [1]. *P. falciparum* malaria alone is responsible for over 800,000 deaths a year, with many of these fatalities occurring in infants in Africa [2].

Artemisinin (ART) and its derivatives are the only remaining drugs that are effective against severe malaria and multi-drug resistant malaria [3]. ART is derived from the Chinese herb *Artemesia annua* (sweet wormwood), which has been used successfully to treat malaria associated fevers since 138 BC [4]. *In vivo*, ART produces fast parasite and fever clearance, is well tolerated, and has an important role in the treatment of severe and cerebral malaria [5]. However, what is not understood about this powerful anti-malarial is that when given alone, there is a high frequency of recrudescence [6], [7]. This high rate of recrudescence has prompted the World Health Organization (WHO) and the International Artemisinin Study Group to recommend that uncomplicated *P. falciparum* malaria be treated with ART or its derivatives in combination with another effective blood schizontocide to delay the selection of resistant strains [8]. While many advocate the use of Artemisinin-based combination therapy (ACT) to delay the development of resistance to the remaining armory of effective drugs, a major impediment to using ACT is its cost [9]. Currently, the cost per treatment is $1.20–2.50 per person, as compared to $0.10–0.20 per person for chloroquine and sulfadoxine-pyrimethamine [9]. Even with ACT, there are still issues with treatment failure and recrudescence of infections [10].

Recrudescence in falciparum malaria is considered a sign of treatment failure and it forces a shift to more potent drugs or drug combinations [11]. Studies by Nakazawa *et al* [11] suggest that treatment failure may be due to either resistant parasite strains that are selected by drug treatment or blood levels of the drug that are insufficient to further suppress the parasite. Those studies also suggest that a small percentage of the parasites may be in a dormant state and are unaffected by drug treatment. When such treatment is discontinued, these parasites resume normal development, resulting in a detectable parasitemia [12], [13], [14]. In a separate *in vitro* study, *P. falciparum* exposed to artesunate (AS) or dihydroartemisinin (DHA) demonstrated that development of early ring-stage parasites was rapidly interrupted, and that these parasites survived in a dormant form for up to 20 days before resuming normal growth [13]. Modeling using the *in vitro* dormancy recovery rates and duration produced treatment failure rates comparable to those reported in the field [15]. More recent microarray studies demonstrate these dormant rings remain in a state of transcriptional arrest at the ring stage before resuming growth approximately 7 days later, further supporting the dormancy hypothesis [14],[Tucker et al 2011, submitted for publication]. However, ART induced dormancy and its role in treatment failure *in vivo* has not been demonstrated and characterized.

In this study, the rodent malaria models *P. vinckei petteri* (non-lethal strain) and *P. vinckei vinckei* (lethal strain) were used to investigate the stage-specific effects of AS on malaria parasites *in vivo*. These parasites were chosen because of their ability to remain synchronous *in vivo*, thus allowing for testing the effect of AS drug action on specific parasite stages. Previous studies have found that the ring and trophozoite stages of *P. v. petteri* were the most susceptible to treatment with the oil-soluble artemether [16]. Our results suggest that ring-stage parasites are the least susceptible to treatment with the water soluble AS and that the effects are dependent upon when the drug is administered. Additionally, dormancy recovery studies reveal that there appears to be a correlation between the number of dormant parasites present following drug exposure and the timing of recrudescence. These studies not only provide a better understanding about the effects that treatment with AS has on specific parasite stages, but also provide insight into possible reasons for recrudescence and subsequent treatment failures.

## Results

### The stage-specific effects of a single dose of AS in *P. v. petteri and P. v. vinckei*

**Figure 1:**
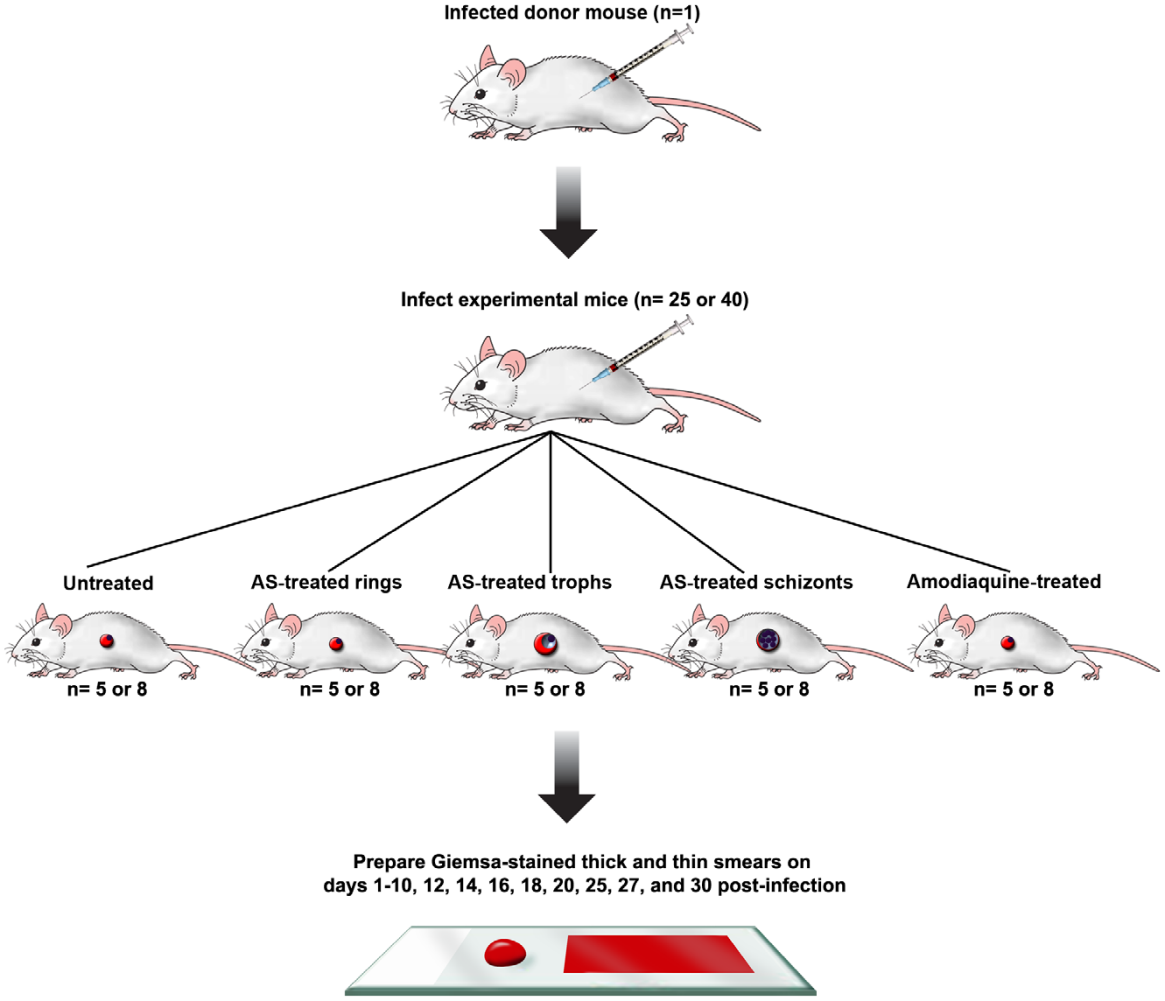
Experimental design for the stage-specific studies. Donor mice were used to infect one batch of 25 or 40 experimental mice with either *P. vinckei vinckei or P. vinckei petteri*, respectively. Mice then were randomly separated into groups of 5 consisting of untreated, artesunate treated (AS) rings, trophozoites (trophs), and schizonts. Amodiaquine treated parasites were included as a positive drug control. Parasitemia were monitored for 30 days.

**Figure 2:**
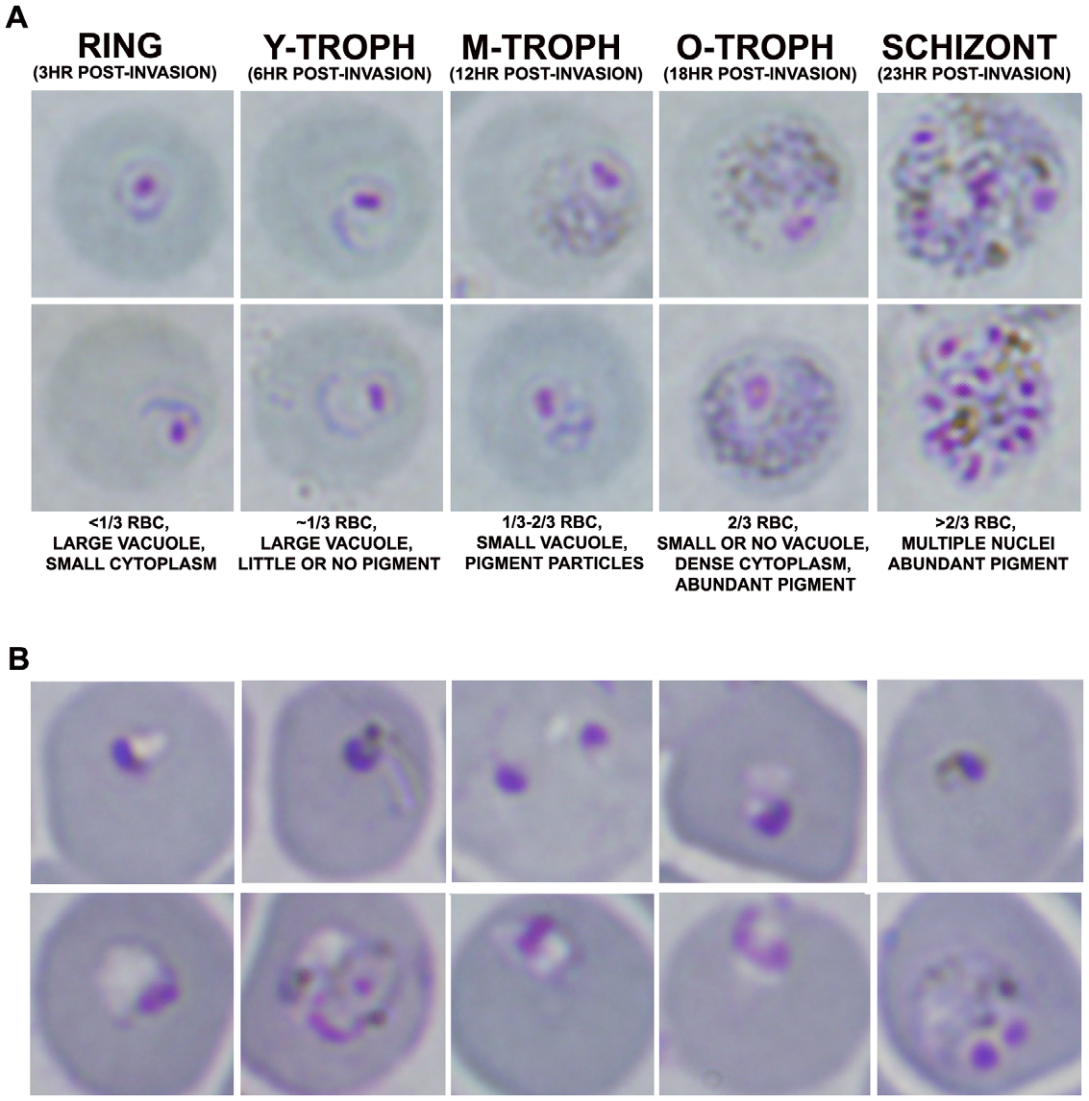
*P. v. vinckei* morphology in mice before treatment and 24 hr following treatment with artesunate. A. Two representative images are shown for each stage of development *in vivo*. Ring-stage (R), young trophozoite (Y-troph), mid-term trophozoite (M-troph), old trophozoite (O-troph), and schizont. (SCH). B. Treated ring-stage parasites (top row) have a condensed nucleus and pyknotic appearance. Older parasite stages treated with artesunate (bottom row) appear to have an absence of hemozoin and loss of membrane integrity.

The goal of these studies was to assess the effects of a single dose of AS (64 mg/kg) on *P. vinckei*. There were five experimental groups infected with either *P. v. petteri* (n = 8 per group) or *P. v. vinckei* (n = 5 per group). To control for variations of infection, one batch of experimental mice were inoculated with parasites at the same time (Figure 1). Mice then were dosed according to a previously published time-line for parasite development [17], [18] to allow stage-specific treatment of rings, trophozoites, and schizonts. Figures 2A and B show examples of what the parasite stages look like before and after treatment. Effectiveness of AS treatment was determined by assessing mean peak parasitemia for the non-lethal strain *P. v. petteri* and survival for the lethal strain *P. v. vinckei*.

#### Rings

**Figure 3:**
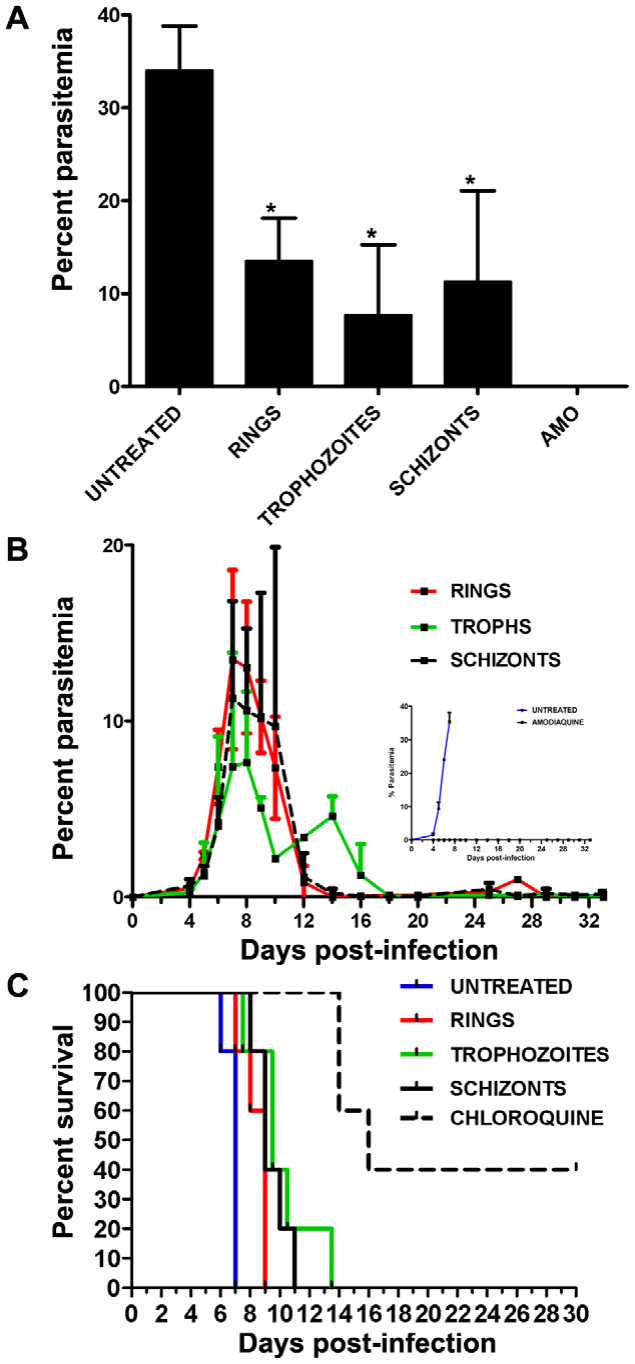
The stage-specific effects of a single dose of AS. A. *Plasmodium vinckei petteri*-infected mice (n = 8 mice per group) were treated with one dose of AS (64 mg/kg). Comparison of peak parasitemias revealed that all treated groups had significantly lower peak parasitemias when compared to the untreated control (Dunnett's multiple comparison test, *p*<0.001). * = statistically significant difference. B. Assessment of average daily parasitemias showed that the ring stage had the highest average parasitemia when compared to treated trophozoites and schizonts. Both the ring and trophozoite groups had a secondary peak on days 27 and 14 PI, respectively. Overall, the treated trophozoites took the longest to clear and had the lowest average parasitemia. Data are means ± SD. C. Mice infected with the lethal strain *P. v. vinckei* (n = 5 mice per group) were treated with one dose of AS (64 mg/kg). The median day of survival for untreated mice, and mice treated when mostly rings, trophozoites, or schizonts were present was 7.0 and 9.0, respectively. No significant difference in survival was observed among the treated groups.

Mice infected with *P. v. petteri* and treated with one dose of AS when rings were present had a mean peak parasitemia of 13.50%±3.16 on day 7 post-infection (PI) (Figure 3A). This peak parasitemia was found to be significantly lower from that observed for the untreated mice on the same day (35.36±14.0, Dunnett's multiple comparison test, *p*<0.001). For the treated rings, the peak on day 7 was followed by parasite clearance on day 14 PI. A recurrence of infection on day 27 PI was due to one mouse with an 8.6% parasitemia that cleared on day 29 PI and remained clear until the end of the experiment on day 30 PI (Figure 3B). In *P. v. vinckei*-infected mice, the untreated group had a median survival of 7.0 days, whereas mice treated with one dose of AS had a median survival of 9.0 days (Figure 3C).

#### Trophozoites

*P. v. petteri*-infected mice treated during the trophozoite stage had a significantly lower mean peak parasitemia of 7.65%±2.114 (Dunnett's multiple comparison test, *p*<0.001) on day 8 PI when compared to the untreated (Figure 3A). One additional peak was observed on day 14 PI (4.6%) due to one mouse with a 40% parasitemia (Figures 3B). By day 18 PI, all mice showed complete clearance of parasitemia until the end of the study. In *P. v. vinckei*-infected mice, the final mouse did not die until day 13.5; however, like the treated rings, groups dosed during the trophozoite stage had a median survival of 9.0 days (Figure 3C).

#### Schizonts

*P. v. petteri*-infected mice treated during the schizont stage had a mean peak parasitemia of 11.30%±2.78 on day 7PI and cleared on day 16 PI (Figure 3A). This peak parasitemia was significantly lower than the untreated (Dunnett's multiple comparison test, *p*<0.001). Low-level recrudescence occurred in the follow-up period of thirty days (Figure 3B). Studies with the lethal strain *P. v. vinckei* that were initiated during the schizont stage resulted in a median survival of 9.0 days (Figure 3C).

Overall, for *P. v. petteri*-infected mice, all treatment groups had significantly lower peak parasitemias when compared to the untreated (Dunnett's multiple comparison test, *p*<0.001). No differences were observed in peak parasitemia between the treated trophozoites and schizonts; however, both stages had significantly lower peak parasitemias than the treated rings (Dunnett's multiple comparison test, *p*<0.05). Mice treated with the control drug amodiaquine remained clear for the duration of the study. For *P. v. vinckei*-infected mice, there were differences between the survival times of the different experimental groups (log rank test, chi sq 13.62, df 1.0, *p*<0.0001). There was no difference in survival following one dose of AS when compared to the untreated groups; however, treatment with one dose of chloroquine (control drug) resulted in a median survival of 16 days, which was significantly higher than the untreated (Dunnett's multiple comparison test, *p*<0.05).

### The effects of repeated dosing on *P. v. vinckei* ring-stage parasites in vivo

#### Consecutive dosing with AS

**Figure 4:**
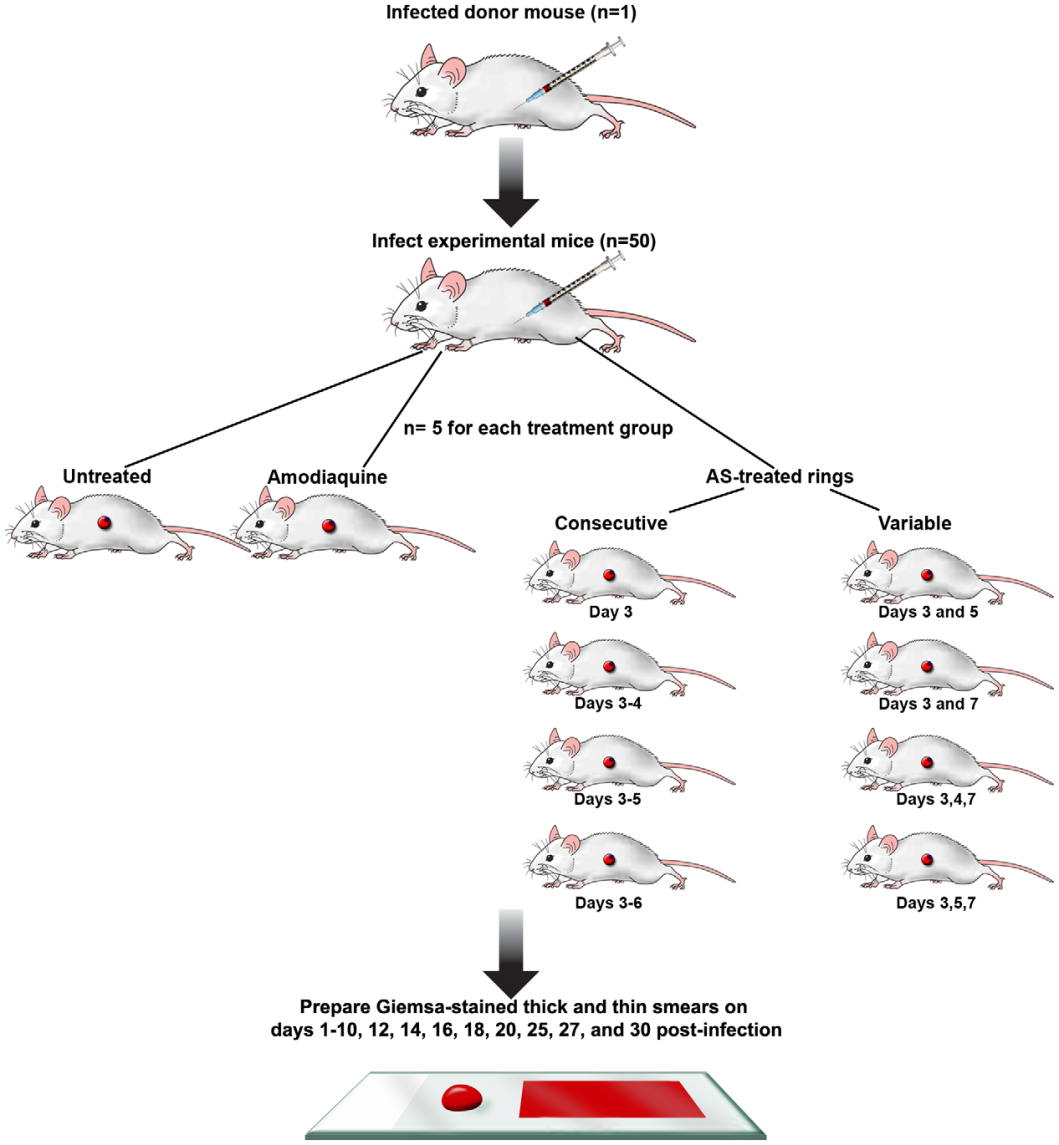
Experimental design for the repeated dosing studies. Donor mice were used to infect one batch of 50 experimental mice with *P. vinckei vinckei*. Mice then were randomly separated into groups of 10 containing 5 mice each and dosed on different days.

**Figure 5:**
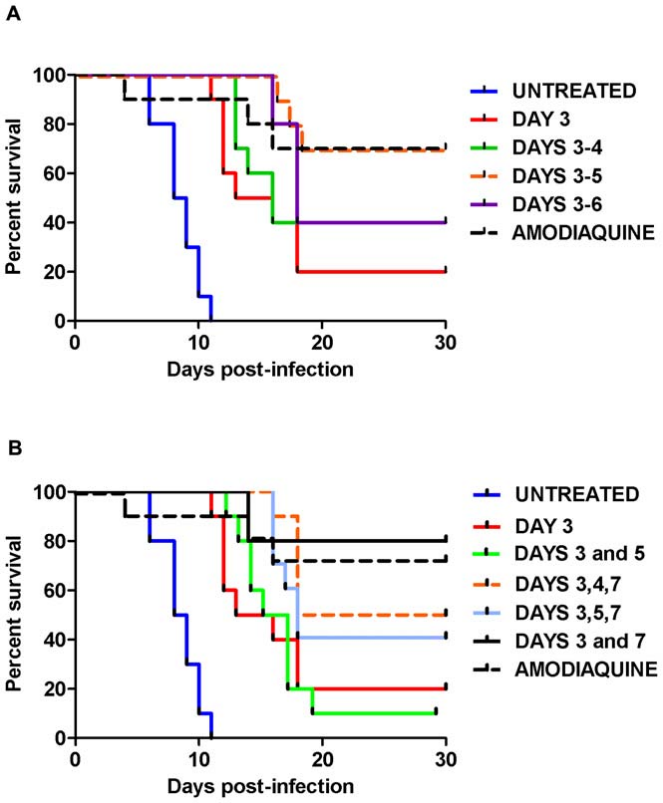
The effects of repeated dosing with AS on *P. v. vinckei*-infected mice. Mice were treated with a total dose of 256 mg/kg of AS and monitored for recrudescence (n = 5 mice per group). A. Mice treated for three or four consecutive days had the highest percent survival (70%) when compared to the untreated (Dunnett's multiple comparison test, *p*<0.01). B. In the variable group, mice treated on days 3 and 7 post-infection (PI) had the highest percent survival (80%) when compared to the untreated (Dunnett's multiple comparison test, *p*<0.05).

To assess the effects of consecutive doses of AS on parasite recrudescence, *P. v. vinckei*-infected mice were treated with a total dose of 256 mg/kg divided over 2–4 days (Table 1). One batch of 50 mice were inoculated IP with *P. v. vinckei* and then randomly divided into ten treatment groups (n = 5 per group; Figure 4). Untreated mice had a median survival rate of 8.5 days, whereas, mice treated with one 256 mg/kg dose of AS had a median survival of 14.5 days (Figure 5A). Following repeated exposure to AS on 2 (128 mg/kg/day) and 4 (64 mg/kg/day) consecutive days the median survival was 16 and 18 days, respectively (Figure 5A). When compared to the untreated, three consecutive days of treatment was the most effective regimen with 70.0% of the mice surviving until day 30 PI (Dunnett's multiple comparison test, *p*<0.01; Figure 5A).

**Table 1:** Artesunate dosing schedule for mice infected with *P. v. vinckei*.

Group	Days Dosed	Schedule of Dosing	Mice per Group	Dose per Day (mg/kg)	Total Dose (mg)	Dosing Rationale
1	Untreated	N/A	5	N/A	N/A	Infection Control; Did not receive treatment
2	3	One day	5	256	256	One day regimen
3	3–4	Two days, consecutive	5	128	256	Two day regimen
4	3–5	Three days, consecutive	5	85.3	256	Standard three day regimen
5	3–6	Four days, consecutive	5	64.0	256	Four day regimen
6	3 and 5	Two days, variable	5	128	256	Two day regimen
7	3,4,7	Three days, variable	5	128	256	Day 4 PT (Day 7 PI)*
8	3,5,7	Three days, variable	5	85.3	256	Day 4 PT (Day 7 PI)*
9	3 and 7	Two days, variable	5	128	256	Day 4 PT (Day 7 PI)*
10	3	Amodiaquine	5	64.0	256	Drug control

There were ten experimental groups. All groups except the untreated received a total dose of 256 mg/kg of AS.

* = Time point chosen because previous *in vitro* studies with *P. falciparum* showed that following treatment with dihydroartemisinin (DHA), dormant ring-stage parasites start to resume growth (*i.e.* normal parasite stages observed) on days 4–5 post-treatment (PT). We wanted to assess how treatment at this time-point would affect parasite recrudescence *in vivo*. N/A = not applicable; PT = post-treatment; PI = post-infection.

#### Variable dosing with AS

The effects of variable dosing on parasite recrudescence were assessed following treatment with a total dose of 256 mg/kg of AS divided equally over 2 or 3 days. Mice treated on days 3 and 5 PI had a median survival of 17 days (Figure 5B). Treatment on days 3, 4, 7 and 3, 5, 7 resulted in a median survival of 24 and 18 days, respectively (Figure 5B). Treatment on days 3 and 7 had the highest survival rate when compared to the untreated, with 80.0% of the mice surviving until day 30 PI (Dunnett's multiple comparison test, *p*<0.05; Figure 5B).

#### Comparison of P. v. petteri and P. v. vinckei

Data from both the consecutive and variable studies were compared to results obtained from a pilot study using the non-lethal *P. v. petteri* (Table 2). In the pilot study, because *P. v. petteri* is a non-lethal strain, peak parasitemia was used as a measure of AS effectiveness. All mice received 64 mg/kg/day of AS which resulted in a different total dose for each treatment group (Table 3). The most effective treatment regimens, because they had the lowest peak parasitemias of 1.21% and 1.58%, were on days 3, 4 and 7 and for 4 consecutive days, respectively (Table 2). Studies with the lethal strain revealed that *P. v. vinckei*-infected mice dosed for 3 consecutive days (85.3 mg/kg/day) had a survival rate of 70% which was significantly higher than the untreated (Dunnett's multiple comparison test, *p*<0.01; Table 2). For the variably dosed groups, mice treated on days 3 and 7 PI (128 mg/kg/day) had the highest survival rate (80%) among all of the treatment groups (Dunnett's multiple comparison test, *p*<0.05; Table 2). Overall, the data show that the day of treatment, rather than the total dose administered, is more important for parasite clearance and/or host survival when treating with AS.

**Table 2:** Experimental survival rates using *P. v. vinckei (Pvv)*, a lethal strain, compared to peak parasitemias from a previous study using *P. v. petteri (Pvp)*, a non-lethal strain.

		*Pvv*-LETHAL		*Pvp*-NON-LETHAL	
Group	Day	Number of doses	% survival	Peak Parasitemia	Day of Peak
1	Untreated	N/A	0%	35.18%	9 PI
2	3	1	20%	12.24%	9 PI
3	3–4	2	40%	6.48%	12 PI
4	3–5	3	**70%** *	4.08%	19 PI
5	3–6	4	40%	**1.58%**	17 PI
6	3 and 5	2	10%	2.40%	10 PI, 27 PI
7	3,4,7	3	50%	**1.21%**	31 PI
8	3,5,7	3	40%	3.41%	7 PI
9	3 and 7	2	**80%** *	7.08%	9 PI
10	Amodiaquine	1	40%	0.70%	19 PI

There were five mice per group in the lethal and non-lethal pilot studies. The most effective regimens in comparison to the untreated group are in bold.

* = *p*<0.05; Dunnett's multiple comparison test; N/A = not applicable.

**Table 3:** Artesunate dosing schedule for mice infected with *P. v. petteri*.

Group	DaysDosed	Schedule of Dosing	Mice per Group	Dose per Day (mg/kg)	Total Dose (mg)	Dosing Rationale
1	Untreated	N/A	5	N/A	N/A	Infection Control; Did not receive treatment
2	3	One day	5	64	64	One day regimen
3	3–4	Two days, consecutive	5	64	128	Two day regimen
4	3–5	Three days, consecutive	5	64	192	Standard three day regimen
5	3–6	Four days, consecutive	5	64	256	Four day regimen
6	3 and 5	Two days, variable	5	64	128	Two day regimen
7	3,4,7	Three days, variable	5	64	192	Day 4 PT (Day 7 PI)
8	3,5,7	Three days, variable	5	64	192	Day 4 PT (Day 7 PI)
9	3 and 7	Two days, variable	5	64	128	Day 4 PT (Day 7 PI)
10	3	Amodiaquine	5	64	64	Drug control

There were ten experimental groups. Each group received 64 mg/kg/day of AS. N/A = not applicable; PT = post-treatment; PI = post-infection.

### Dormant parasite recrudescence

*In vivo* dormant parasite recrudescence studies were performed with *P. v. vinckei* to determine if the number of dormant parasites present in the vertebrate host directly correlates with observance of recrudescence. Additionally, given that the spleen plays a role in clearance of malaria parasites [7], [19], [20], differences in clearance of dormant parasites in intact and asplenic mice were evaluated. In this study, donor mice were infected with *P. v. vinckei* and when parasitemia were >10.0% with mostly rings present, mice were treated with AS. Twenty-four hours post-treatment, blood was harvested from mice via cardiac puncture and smears were prepared. Analysis of Giemsa-stained blood smears revealed that a majority of the treated rings were dormant, as defined by small rounded forms with a dark staining nucleus (Figure 2B; top row). Experimental mice, intact (n = 30) and splenectomized (n = 30), were injected with a range of dormant parasites (40 to 4×10^6^ dormant parasites/100 µl) and rate of recrudescence (time to reach 5.0% parasitemia) was compared between the two groups using Giemsa-stained thin smears. Intact (n = 10) and asplenic mice (n = 10) injected with untreated ring-stage parasites (40 and 4×10^6^) were included as controls. Percent survival was determined for the entire 30 day assay (Table 4).

**Table 4:** Survival and time to recrudescence following inoculation of artesunate-treated *P. v. vinckei* parasites.

		% Survival		Time to reach>5% Parasitemia (days)	
Group	Parasites per 100 µl	Intact (n = 5)	Asplenic (n = 5)	Intact (n = 5)	Asplenic (n = 5)
Untreated	4×10^6^	0	0	3.2±1.1	4.0±2.3
Untreated	4×10^1^	20	0	6.0±4.4	5.8±1.1
Dormant	4×10^6^	0	0	6.6±1.3	7.6±1.5
Dormant	4×10^5^	20	0	5.4±3.1	7.0±1.0
Dormant	4×10^4^	0	0	8.0±0.0^a^	8.2±0.4
Dormant	4×10^3^	0	0	8.6±0.5^a^	5.8±5.3
Dormant	4×10^2^	20	20	8.4±4.8^a^	4.4±6.1
Dormant	4×10^1^	100	40	N/A	N/A

There were a total of 16 experimental groups with five mice per group (n = 5). Untreated *P. v vinckei* parasites were injected into experimental mice using quantities from the lowest and highest dose in the dormant parasite dilution series.

a Means significantly different to untreated 4×10^6^, *p*<0.05; Dunnett's multiple comparison test. N/A = never reached greater than 5%. Data are means ±SD.

**Figure 6:**
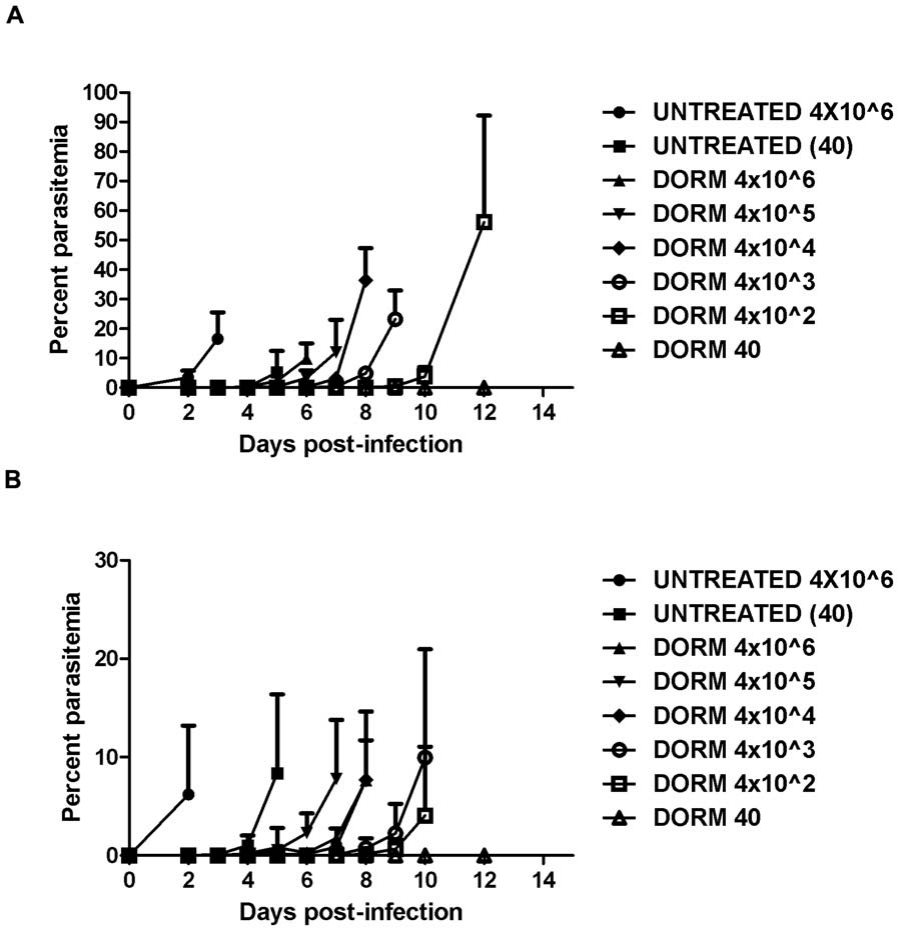
Rate of dormant parasite recrudescence in intact and asplenic mice. Rate of recrudescence was defined as the time to reach >5.0% parasitemia following injection of normal untreated or dormant parasites (n = 5 mice per group). Data are shown as mean total parasitemia +SD versus time. A. In the intact group, rate of recrudescence from dormancy occurred in a dose-dependent manner, with the highest dilution recovering faster. B. In the asplenic group, rate of recrudescence occurred in a dose-dependent manner from 4×10^4^ to 4×10^6^, after which the separation is not as well-defined. DORM = dormant parasites.

**Figure 7:**
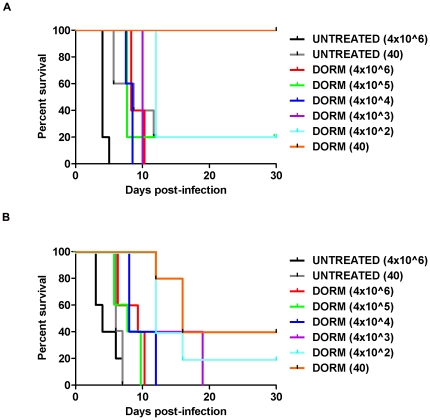
Intact and asplenic mouse survival following exposure to dormant *P. v. vinckei* parasites. Mice were exposed to varying numbers of untreated and dormant (dorm) parasites (n = 5 mice per group). A. Only 20.0% of intact mice exposed to 40 normal untreated parasites, and 4×10^2^ and 4×10^5^ dormant parasites survived until day 30 post-infection (PI). All mice exposed to 40 dormant parasites survived unto day 30 PI. B. Asplenic mice exposed to 4×10^2^ and 40 dormant parasites had a 20.0% and 40.0% survival rate, respectively.

**Figure 8:**
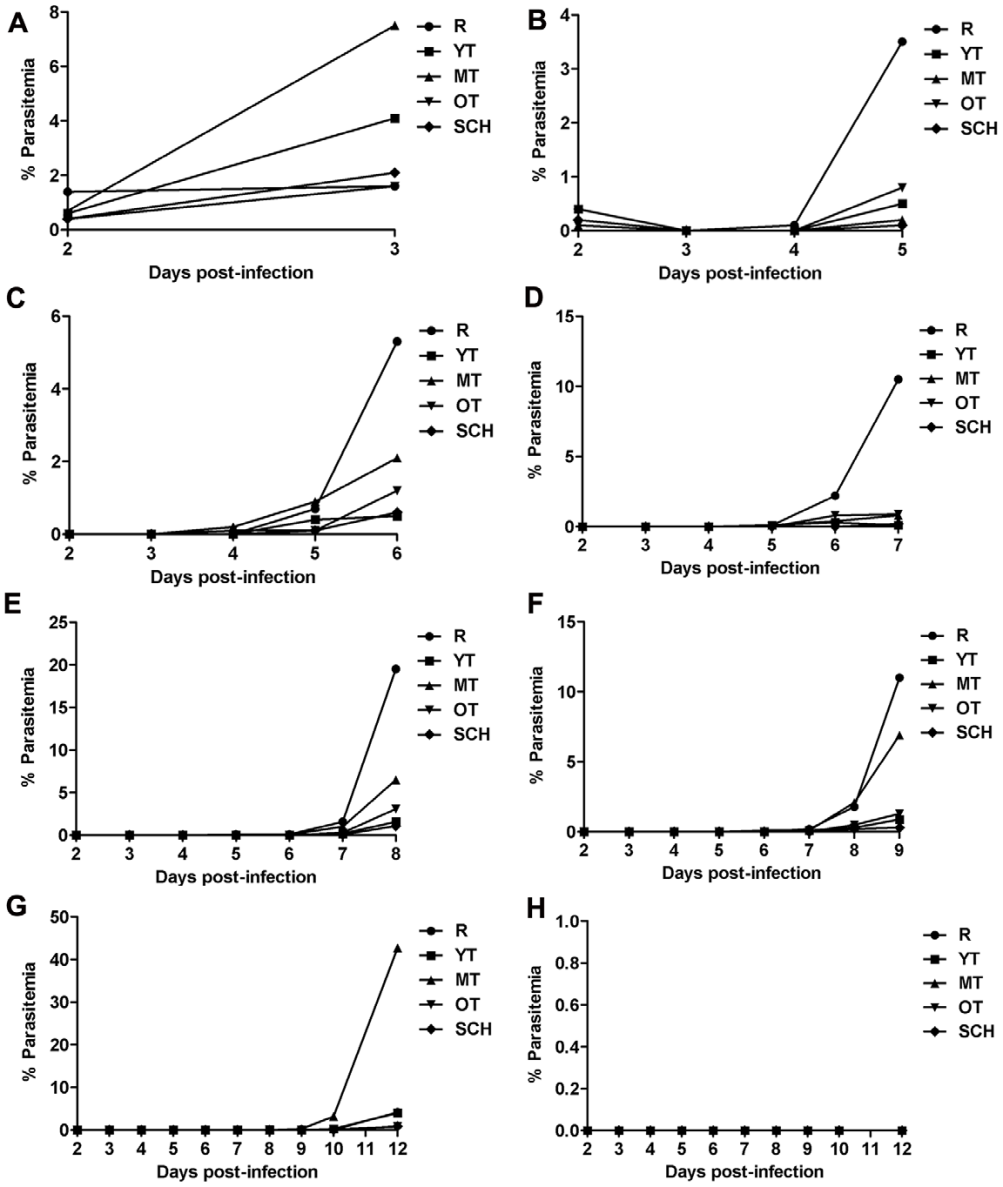
Primary stages present during dormant parasite recrudescence in intact mice. The average parasitemia versus time in relation to the number of dormant parasites are shown in the figure (n = 5 mice per group). Rate of recrudescence from dormancy was defined as the average time to >5.0% parasitemia. In all treatment groups, with the exception of the untreated 4×10^6^, rings are the predominant stage present as parasites begin to recover. A (untreated 4×10^6^), B (untreated 4×10^1^), C (dorm 4×10^6^), D (dorm 4×10^5^), E (dorm 4×10^4^), F (dorm 4×10^3^), G (dorm 4×10^2^) andH (dorm 4×10^1^). Ring (R), young trophozoite (YT), mid-term trophozoite (MT), old trophozoite (OT), and schizont (SCH).

Results revealed that, for intact and asplenic mice, there is a positive correlation between the numbers of dormant parasites present and when parasite recrudescence occurs (Figures 6A and B). Overall, the survival curves of the intact (log rank test, chi sq 14.45, df 1.0, *p*<0.0001) and asplenic (log rank test, chi sq 28.87, df 1.0, *p*<0.0001) mice were significantly different. The average survival of the intact mice and asplenic mice was 20.0% and 7.5%, respectively (Figures 7A and B). However, mice injected with 4×10^1^ dormant parasites, were the only group to have a significantly higher survival rate (100%) when compared to mice infected with 4×10^6^ untreated rings (Dunnett's multiple comparison test, *p*<0.05; Table 4). Additionally, as parasites began to recover, ring-stage parasites were the predominant stage present, suggesting that it is the activated, formally dormant rings that are responsible for the recrudescence observed following artemisinin treatment (Figures 8 A–H).

## Discussion

### Stage-specific effects

Based on results from previous *in vitro* assays, we hypothesized that treated ring-stage parasites would have a delay in the pre-patent period due to the formation of dormant ring forms, trophozoites would be killed, and schizonts would be delayed in the time to reach the euthanasia end point of 40.0% parasitemia. Therefore, in these studies the stage-specific effects of a single dose of AS in two synchronous rodent malaria models, *P. v. petteri* (non-lethal) and *P. v. vinckei* (lethal), were examined.

Results showed that there was no difference in average parasitemia among the treated groups in the stage-specific studies. However, the average peak parasitemia data suggest that the trophozoite stage was most susceptible to treatment with AS, followed by the schizonts stage. Both stages were more susceptible to AS treatment than ring-stage parasites (Figure 3A). These findings differ from those found in studies by Caillard et al [16], which indicated that rings and young trophozoites were the most susceptible to treatment with an artemisinin derivative. Possible reasons for the differences seen in this study versus the Caillard study are that they used arteether, an oil-soluble compound, which was injected subcutaneously and as a result had a prolonged exposure in the blood stream rather than the more rapid elimination of AS following oral administration [21]. More importantly, Caillard et al [16] only followed the parasitemias for 48 hours, whereas we saw recrudescence well after this time period. Another possible reason for the differences in these studies is the self-clearing phenotype exhibited by *P. v. petteri*. Studies have shown that untreated infections with *P. v. petteri* peaked on day 10 PI and spontaneously cleared on day 20 PI [22]. Although untreated infections in this study reached greater than 40% parasitemia before the experimental groups, it is important to note that the peak parasitemia occurred on the same day. It is possible that we would have observed a similar trend in our untreated groups as the experimental groups had they not been euthanized at the cutoff parasitemia of 40.0%.

Our results with *P. v. petteri* appear to be similar to an uncomplicated malaria case in which the patient has a parasitemia, but does not exhibit the pathology associated with severe disease. Perhaps the lingering parasites are suppressed by the action of AS long enough for the host immune response to be activated and clear the parasitemia. These factors make this model ideal for studying the effects of drug treatment in conjunction with a host immune response [23].

This is not the case for the lethal strain *P. v. vinckei*, to which immunity can only be attained from prior exposure [18]. To further evaluate the stage-specific effects of AS in the absence of a rapid host-immune response, the synchronous rodent malaria parasite *P. v. vinckei* was used. Previous studies used *P. v. vinckei* to examine immune mechanisms that must act quickly to protect against death [18], [24]. As expected, our studies showed that with this lethal strain, regardless of the stage of development, a single dose of AS is not sufficient to control a rise in parasitemia. This is not surprising, as others have found that treatment with AS does not cure rodent malaria infections [25]. Additionally, recrudescence also has been observed in malaria-infected patients treated with therapeutic (*i.e.* curative) doses of AS for multiple days [8], [26]. Accordingly, additional experiments were conducted to assess how multiple doses of AS would impact the development of *P. v. vinckei* ring-stage parasites.

### The effects of repeated dosing

In this study, we characterized the effects of different dosing regimens with AS using the rodent malaria model *P. v. vinckei*. Others have shown that artemisinin derivatives induce dormancy in ring-stage parasites [13] and our goal was to determine the effects of repeated dosing on dormancy. We hypothesized that in dormant ring form, parasites would not be affected by additional drug treatment. Additionally, because previous *in vitro* studies have shown that dormant rings begin to recrudesce around day 7–9 PT (3–4 erythrocytic cycles PT), we wanted to determine how dosing on day 7 PI (*i.e.* 4 erythrocytic cycles PT) *in vivo* would affect treatment outcome. Therefore, for this study, we compared a standard three-day regimen with variable treatment times to determine if the total dose administered or day of drug administration would affect parasite clearance and recrudescence.

All mice received the same total dose of 256 mg/kg (Table 1) and results indicate that one single dose of AS (256 mg/kg) is not sufficient to control a rise in parasitemia. Overall, mice that were treated for 3 consecutive days (85.3 mg/kg/day) and on days 3 and 7 PI (128 mg/kg/day) had the highest survival rates (Table 2). In comparison, a pilot study using the non-lethal strain *P. v. petteri* showed that treatment for 4 consecutive days (256 mg/kg total) and on days 3,4,7 (192 mg/kg total) were the most effective (Table 2). Interestingly, although mice in the non-lethal study received different total doses (Table 3), overall the data suggest that the day of treatment with AS is more important than the total dose administered. Additionally, it appears that in both strains, dosing on day 4 PT (day 7 PI) was the most effective for controlling parasitemia. A possible explanation for this finding is that, on day 4 PT, dormant parasites may be starting to recover and drug administration at this time arrests newly activated ring-stage parasites. Additionally, Teuscher et al [13] showed that only a fraction of dormant ring stages survive, therefore it is likely that treating with AS on day 4 PT was sufficient to eradicate the remaining parasites.

### Dormant parasite recrudescence

Previous studies by Teuscher et al [13] have shown that following treatment with AS, *P. falciparum* ring-stage parasites become dormant and approximately 0.001–1.313% recover to resume growth depending on the strain. In those studies, parasite recovery from dormancy was found to be dose-dependent. To determine if this same phenomenon occurs *in vivo*, dormant parasite recovery (time to reach >5.0% parasitemia) was assessed using *P. v. vinckei*. Results showed that dormant forms, similar to those found *in vitro*, are present in treated mice and that the day of parasite recovery is positively related to the number of dormant parasites present in intact mice; however, this was more variable in asplenic mice. Based on our experimental design we cannot definitively calculate recovery rates. However, we can assume that because groups injected with 40 parasites did not recover, that the recovery rates are in the order of 1 in 400 dormant parasites (0.25%) which are comparable to those found in previous studies [13].

#### Role of the spleen

Approximately 5.0% of the blood that is circulated through the body goes to the spleen and on average, RBCs cross the spleen every 20 minutes [27]. When patients are infected with malaria, intra-erythrocytic parasite development results in remodeling of both infected and non-infected RBCs [20]. The spleen filters these altered RBCs and by doing so may increase anemia thereby, reducing high parasite loads associated with severe malaria infections [20]. Studies by Newton et al [28] suggest that this splenic-pitting is responsible for a majority of the parasite clearance that is seen in patients following artemisinin treatment.

Asplenic individuals living in falciparum endemic regions, exhibit more frequent fevers, higher parasitemias, and longer parasite clearance times following treatment with antimalarials such as artemisinin [29], [30]. Studies using *P. berghei*-infected mice, found that animals with full splenectomies had parasitemias double those found in intact and partially splenectomized mice [31]. Additionally, when Moore et al [32] assessed parasite clearance in DHA-treated intact and asplenic mice, it was found that the capacity to clear parasites was reduced in the asplenic population. Similarly, our studies showed that dormant parasites required less time to recover when injected into splenectomized mice and that overall survival of asplenic mice was lower than in the intact group. These data confirm the role of the spleen in clearance of parasites following treatment, yet also demonstrate the possible evasion of spleen pitting of the dormant rings. Additional studies of dormant rings and spleen clearance are warranted.

### Summary

We described for the first time the presence of dormant asexual forms *in vivo* following treatment with AS. It is unclear whether this phenomenon is conserved solely to AS; therefore, future studies should investigate if other artemisinin derivatives and antimalarials produce these dormant forms and if recrudescence is observed. Additionally, because of the differences in response between the non-lethal and lethal strains, future studies should include a comparison of the number of dormant parasites that are produced by a non-lethal versus a lethal stain. This would help determine if there is a correlation between the number of dormant parasites, the severity of disease, and the effectiveness of treatment. If there is a difference, perhaps a change in the recommended treatment strategies needs to be addressed based on the type of infection presented. Finally, studies investigating the mechanism behind dormancy should also be conducted. These findings strengthen the need to use ACT to reduce the possibility of recrudescence following artemisinin monotherapy and provide valuable information for optimal dosing regimens in the treatment of malaria that may prove to be beneficial for preventing parasite recrudescence.

## Materials and Methods

### Ethics statement

This study was conducted in compliance with the Guide for the Care and Use of Laboratory Animals of the National Research Council for the National Academies. The protocol was approved by the University of South Florida Institutional Animal Care and Use Committee (IACUC Protocol Number: R 3655). The numbers of animals used were the minimum required for obtaining scientifically valid data. Experimental procedures were designed to minimize harm and included predefined parasitological endpoints to avoid unnecessary suffering.

### Animals and Parasites

All mice used in these experiments were female ICR outbred mice (average weight was approximately 21g) obtained from Harlan (Fredrick, MD). The rodent malaria parasites *P. v. petteri* 4BS2 (Malaria Research and Reference Reagent Center, Manassas, VA) and *P. v. vinckei* ATCC 30091 (American Type Culture Collection, Rockville, MD) were used for these studies. These parasites were chosen because they remain highly synchronous *in vivo*, allowing for treatment of specific parasite stages. *P. v. petteri* results in a non-lethal infection and is often used to study immune mechanisms [23]. *P. v. vinckei*, which causes a lethal infection, is often used to study pathogenesis and for chemotherapy studies [23]. Both strains are chloroquine sensitive.

### Drugs

The AS (AS), chloroquine, and amodiaquine (AMO) used in these studies were obtained through Sigma (St. Louis, MO). The drugs for these studies were reconstituted to the required dose needed for each experiment in ddH_2_0 or 0.5% hydroxyethylcellulose (HEC). Drugs were administered with an oral gavage cannula.

### Inoculation of malaria parasites

Donor mice were infected intra-peritoneally (IP) with *P. v. petteri* or *P. v. vinckei* parasites obtained from cryopreservation. Blood from donor mice was obtained via cardiac puncture, erythrocytes were counted, and parasitemia determined. Different parasite concentrations were tested in pilot studies and it was determined that 1×10^6^ *P. v. petteri* and 1×10^3^ *P. v. vinckei* parasites were optimal for these studies. Experimental mice were infected IP with 0.1 ml of parasites using a 1.0 ml syringe and a 26 gauge needle.

### Determination of parasitemia

Parasitemias were monitored by daily microscopic examination of methanol fixed blood smears from mice tail veins. Smears were stained with 20.0% Giemsa for 20 minutes, rinsed, and dried prior to viewing. Approximately 600 red blood cells (RBC) per experimental slide were counted and parasitemias, as well as stages present on examination, were recorded. Mice were euthanized when parasitemias were ≥40.0%, according to IACUC regulations.

For the repeated dosing studies, parasitemias were monitored daily by fluorescence activated flow sorting using SYBR green, as previously described [33]. Briefly, one drop of mouse tail blood was collected into 50 µl of heparin (10 IU) in a 96-well microtiter plate. Samples then were incubated in the dark with 30 µl of SYBR Green I (1∶1000 in PBS) for 30 minutes at 37°C. Following the incubation, 150 µl of PBS was added to each well and the samples were mixed. Samples then were diluted 1∶10 in PBS and transferred in triplicate to a new 96-well plate for flow analysis. The Accuri C6 Flow Cytometer (Accuri Cytometers Inc., Ann Arbor, MI) was used to acquire 100,000 events per sample. Initial gating was carried out using unstained, non-infected and stained, non-infected mouse blood to account for erythrocyte auto-fluorescence and SYBR green background staining, respectively. To confirm flow cytometry readings, parasitemias also were calculated via microscopic examination as described previously.

### In vivo studies

To assess the effects of AS on specific malaria stages in a non-lethal and lethal strain, *P. v. petteri* and *P. v. vinckei* were used, respectively. Timing for drug administration was based on previously published studies [16], [17]. Figure 2A illustrates the characteristics and stages present during dosing.

#### The stage-specific effects of a single dose of AS on recrudescence

For this study one batch of 40 mice were infected IP with *P. v. petteri* and then separated into five treatment groups (n = 8 mice per group; Figure 1). Groups consisted of untreated rings (infection control), as well as mice treated with one dose of AS (64 mg/kg) when rings, mid-term trophozoites, or schizonts were highly predominant. For the drug control, a group of mice were treated during the ring stage with 64 mg/kg AMO. Treatment was initiated on day 3 PI when the mean parasitemia was ≥0.32%. Slides were prepared on days 1–10 PT and then every other day until day 20, followed by days 25, 27, and finally day 30 PT. This study was performed three times with the same treatment group comparisons performed within each replicate.

For the *P. v. vinckei* experiment, one batch of 25 mice were inoculated IP. There were five groups (n = 5 mice per group) randomly divided into treated rings, mid-term trophozoites, and schizonts. Control groups consisted of untreated and CQ treated (64 mg/kg; drug control) mice.

#### The effects of repeated dosing on P. v. vinckei ring-stage parasites in vivo

One possible outcome of treatment with AS is that when parasites enter dormancy they will be less affected by repeated exposure to the drug. To test this theory, *P. v. vinckei* ring-stage parasites were repeatedly exposed to AS either on consecutive or variable days (Table 1). One batch of 50 mice were infected with *P. v. vinckei* and then separated into ten groups (n = 5 mice per group; Figure 4). In a pilot study, *P. v. petteri* ring-stage parasites treated with four doses of 64 mg/kg of AS resulted in a significant increase in survival when compared to the untreated control. Therefore, all mice in this study were treated with a total of 256 mg/kg of AS. Group 4 represents a normal three-day regimen of AS and groups 7–9 were chosen to determine if dosing on day 4 PT (day 7 PI), when dormant parasites are expected to recrudesce, would lengthen the time to reach the euthanasia end point of ≥40% parasitemia when compared to the untreated control. Control groups consisted of untreated and amodiaquine treated (64 mg/kg; drug control) mice. This study was performed twice with the same treatment group comparisons performed within each replicate.

#### Dormant parasite recrudescence

Parasite recrudescence from dormancy (time to reach >5% parasitemia) was assessed *in vivo* using *P. v. vinckei*. To avoid parasite clearance by the spleen and maximize the number of dormant parasites, female ICR splenectomized mice (15–20 g) were used. Infections were initiated as described previously and when the parasitemia was >10% rings, mice were treated with AS (256 mg/kg). Twenty-four hours PT, dormant parasites were collected via cardiac puncture, serially diluted (4×10^6^ to 40 dormant parasites/100 µl), and injected IP (0.1 ml) into experimental mice (n = 30 intact and 30 asplenic). Control mice (n = 10 intact and 10 asplenic) were injected with the lowest (40) and the highest dilution (4×10^6^) of normal untreated rings. For these studies, both asplenic and intact mice were used, so that a comparison between recrudescence rates could be observed. To minimize batch effects, all experimental mice were infected on the same day. There were a total of 16 groups containing five mice each (Table 4).

### Statistical analysis

All statistical analyses were carried out using GraphPad Prism 5 (GraphPad Software Inc, La Jolla, CA, USA). All values are expressed as mean and SD. A *p*-value of ≤0.05 was considered statistically significant. Data were analyzed using one-way ANOVA with Dunnett's post-hoc test when comparing treated group means to an untreated control. The log-rank test was performed to compare survival rates.
